# Supplementary material for: RNAi-based knockdown of candidate gut receptor genes altered the susceptibility of Spodoptera frugiperda and S. litura larvae to a chimeric toxin Cry1AcF
Source: PeerJ. 2023 Jan 24;11:e14716. doi: 10.7717/peerj.14716 (PMC9881468; doi:10.7717/peerj.14716)
Supplement: Supplemental Information 1 [file peerj-11-14716-s001.pdf]

Supplementary Table 1. Oligonucleotides used for RNAi and RT-qPCR analysis in *S. frugiperda*. T_m = 60°C

**Gene**	**Primer orientation**	**Primer sequence (5'-3')**	**Purpose**	**PCR efficiency (%)**	**Standard curve R^2**
CAD	Sense	GAGCTCGTGTTGAACGACGGTGACAA [a]	dsRNA synthesis	NA	NA
	Antisense	AAGCTTGACCCTGTACTGCTCCTGTT [b]			
ABCC2	Sense	GAGCTCTGCTGGCAAGTCATCTCTGA	dsRNA synthesis	NA	NA
	Antisense	AAGCTTCTTTTGGATCAGAGCGTCGG			
ALP1	Sense	GAGCTCGACTCGCTCATTGTGGTCAC	dsRNA synthesis	NA	NA
	Antisense	AAGCTTACAAGGAGAGAGAGCAGCAG			
APN	Sense	GAGCTCCTTGCTACGATGAACCTGGC	dsRNA synthesis	NA	NA
	Antisense	AAGCTTCCAGTTTTCCATAGCACCGG			
GFP	Sense	GAGCTCGCAGAGCGAGGTATGTAGGC [a]	dsRNA synthesis	NA	NA
	Antisense	AAGCTTCTGCCTCGGTGAGTTTTCTC [b]			
CAD	Sense	GAGAGCTGAGGGTCACTTGG	RT-qPCR	104.8	0.952
	Antisense	ATATCGGCCAGTTGTCGTTC			
ABCC2	Sense	GTCAGGGCGAGTACTTCGAG	RT-qPCR	102.9	0.921
	Antisense	CACGCTCCAATAAGACAGCA			
ALP1	Sense	CAAGGAGAGCGTGAAGAAGG	RT-qPCR	100.6	0.905
	Antisense	TCTGAGCGTTCACGCAATAC			
APN	Sense	CTATACGCGAGCGTTGAACA	RT-qPCR	106.4	0.922
	Antisense	TGAGATGCATACGGAGCAAG			
RPS3	Sense	ACAGAGTGTGCTCGGAGAGA	RT-qPCR	107.0	0.904
	Antisense	GGCAAGACCTCCAATGAGTT			

[a] underlined sequence indicates *Sac*I endonuclease site, [b] underlined sequence indicates *Hind*III endonuclease site, NA-not applicable.
