# Supplementary material for: RNAi-based knockdown of candidate gut receptor genes altered the susceptibility of Spodoptera frugiperda and S. litura larvae to a chimeric toxin Cry1AcF
Source: PeerJ. 2023 Jan 24;11:e14716. doi: 10.7717/peerj.14716 (PMC9881468; doi:10.7717/peerj.14716)
Supplement: Supplemental Information 2 [file peerj-11-14716-s002.pdf]

Supplementary Table 2. Oligonucleotides used for RNAi and RT-qPCR analysis in *S. litura*. T_m = 60°C

Gene	Primer orientation	Primer sequence (5'-3')	Purpose	PCR efficiency (%)	Standard curve R^2
CAD	Sense	GAGCTCAGTGTTGGACAGGGATGGAG [a]	dsRNA synthesis	NA	NA
	Antisense	AAGCTTTGGCCTGACGGTTAGTTCAT [b]			
ABCC2	Sense	GAGCTCTGCCACTTCAAGCTGGTCTA	dsRNA synthesis	NA	NA
	Antisense	AAGCTTGAATGTCTGAACGCTCGTCC			
ALP1	Sense	GAGCTCAAGCGCGAAGAAATGGATCC	dsRNA synthesis	NA	NA
	Antisense	AAGCTTTCGCGTTGTTGTCACGATAC			
APN	Sense	GAGCTCTACTACGCCACCACACAGTT	dsRNA synthesis	NA	NA
	Antisense	AAGCTTAAGTCAGGCAGCGCAATATG			
GFP	Sense	GAGCTCGCAGAGCGAGGTATGTAGGC [a]	dsRNA synthesis	NA	NA
	Antisense	AAGCTTCTGCCTCGGTGAGTTTTCTC [b]			
CAD	Sense	GCTGGCTCTGGCTAATATCG	RT-qPCR	105.7	0.992
	Antisense	CAAACTGTTGGACACCATCG			
ABCC2	Sense	AACGTCAGCTGGTGTGTCTG	RT-qPCR	105.2	0.911
	Antisense	CAGCAAGATGTAGGGGTGGT			
ALP1	Sense	CGAATACATTTGGCATCGTG	RT-qPCR	102.6	0.945
	Antisense	GGATTGCCACCTCAGTCATT			
APN	Sense	CATCAACACAAACCCTGTCG	RT-qPCR	104.8	0.902
	Antisense	TGATGGCCATGTTGGAGTAA			
β-actin	Sense	GCCATGTACGTCGCCATC	RT-qPCR	100.5	0.914
	Antisense	CAGGTAGTCTGTGAGGTCGC			
GAPDH	Sense	GACCAACTGTCTCGCTCCTC	RT-qPCR	105.0	0.908
	Antisense	CCAGAGGGTCCATCAACAGT			

[a] underlined sequence indicates *Sac*I endonuclease site.
[b] underlined sequence indicates *Hind*III endonuclease site.
NA, not applicable.
