# Supplementary material for: RNAi-based knockdown of candidate gut receptor genes altered the susceptibility of Spodoptera frugiperda and S. litura larvae to a chimeric toxin Cry1AcF
Source: PeerJ. 2023 Jan 24;11:e14716. doi: 10.7717/peerj.14716 (PMC9881468; doi:10.7717/peerj.14716)

Supplementary Figure 2. Tissue-specific expression patterns of Cry receptor encoding genes in different tissues of *S. frugiperda* fourth-instar larvae. Fold change in expression of a candidate gene in different tissues (fat body (FB), foregut (FG), midgut (MG), hindgut (HG) and Malpighian tubules (MT)) was quantified in relation to the gene's expression in head (H) tissue (value set at 1). Significant differential expression is indicated by different letters ($P < 0.01$, Tukey's HSD test). *S. frugiperda rps3* gene was used as the internal reference. Each bar represents the mean fold change value ± SE of qPCR runs in five biological and three technical replicates.

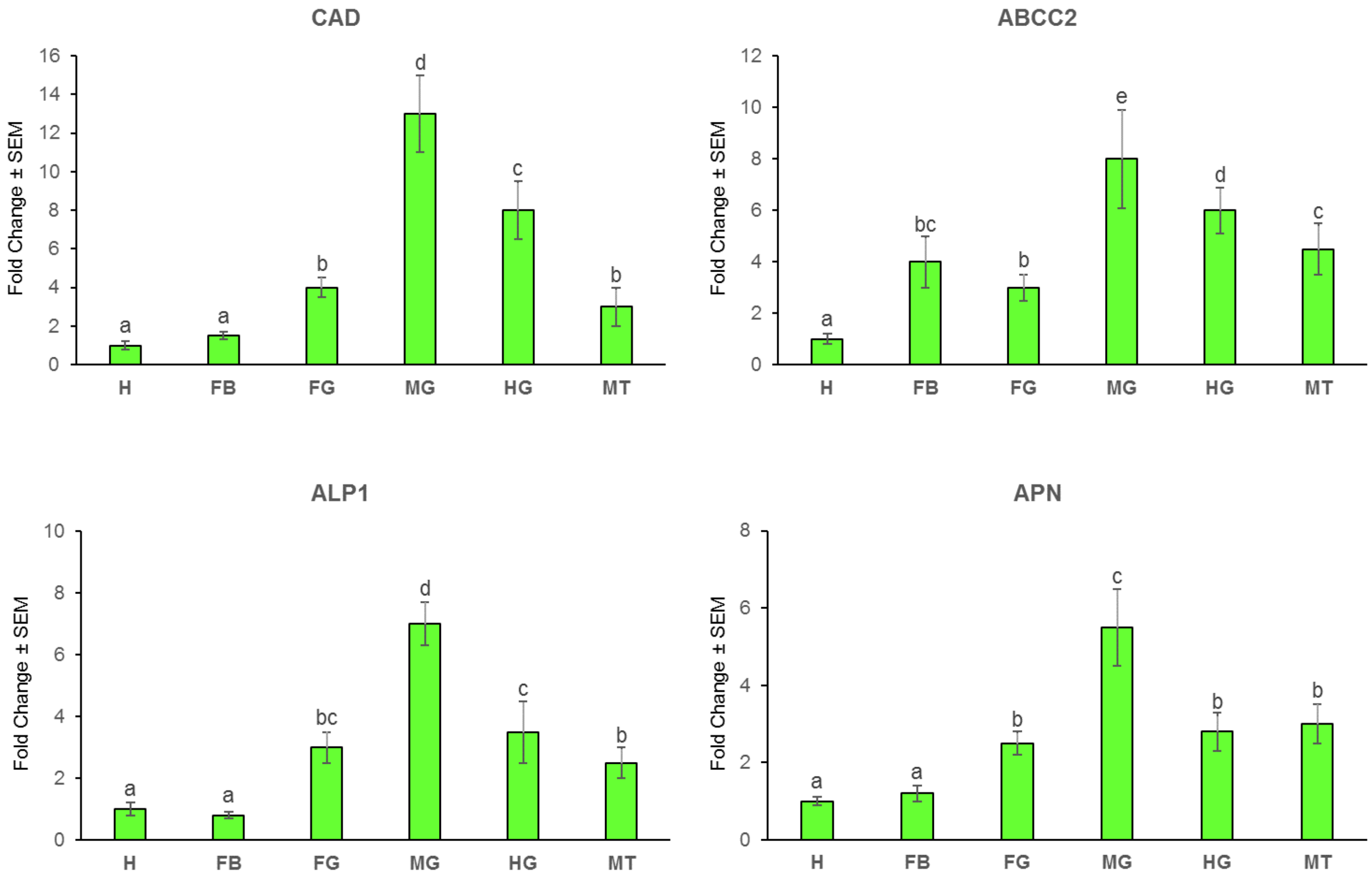

Supplement: Supplemental Information 4 — Fold change in expression of a candidate gene in different tissues (fat body (FB), foregut (FG), midgut (MG), hindgut (HG) and Malpighian tubules (MT)) was quantified in relation to the gene’s expression in head (H) tissue (value set at 1). Significant differential expression is indicated by different letters (P < 0.01, Tukey’s HSD test). S. frugiperda rps3 gene was used as the internal reference. Each bar represents the mean fold change value ± SE of qPCR runs in five biological and three technical replicates. [file peerj-11-14716-s004.pdf]
