# Supplementary material for: RNAi-based knockdown of candidate gut receptor genes altered the susceptibility of Spodoptera frugiperda and S. litura larvae to a chimeric toxin Cry1AcF
Source: PeerJ. 2023 Jan 24;11:e14716. doi: 10.7717/peerj.14716 (PMC9881468; doi:10.7717/peerj.14716)

Supplementary Figure 3. Stage-specific expression patterns of Cry receptor encoding genes in different life stages of *S. litura*. Fold change in expression of a candidate gene in different developmental (second-, third-, fourth- and fifth-instar) stages was quantified in relation to the gene's expression in first-instar stage (value set at 1). Significant differential expression is indicated by different letters ($P < 0.01$, Tukey's HSD test). *S. litura GAPDH* gene was used as the internal reference. Each bar represents the mean fold change value ± SE of qPCR runs in five biological and three technical replicates.

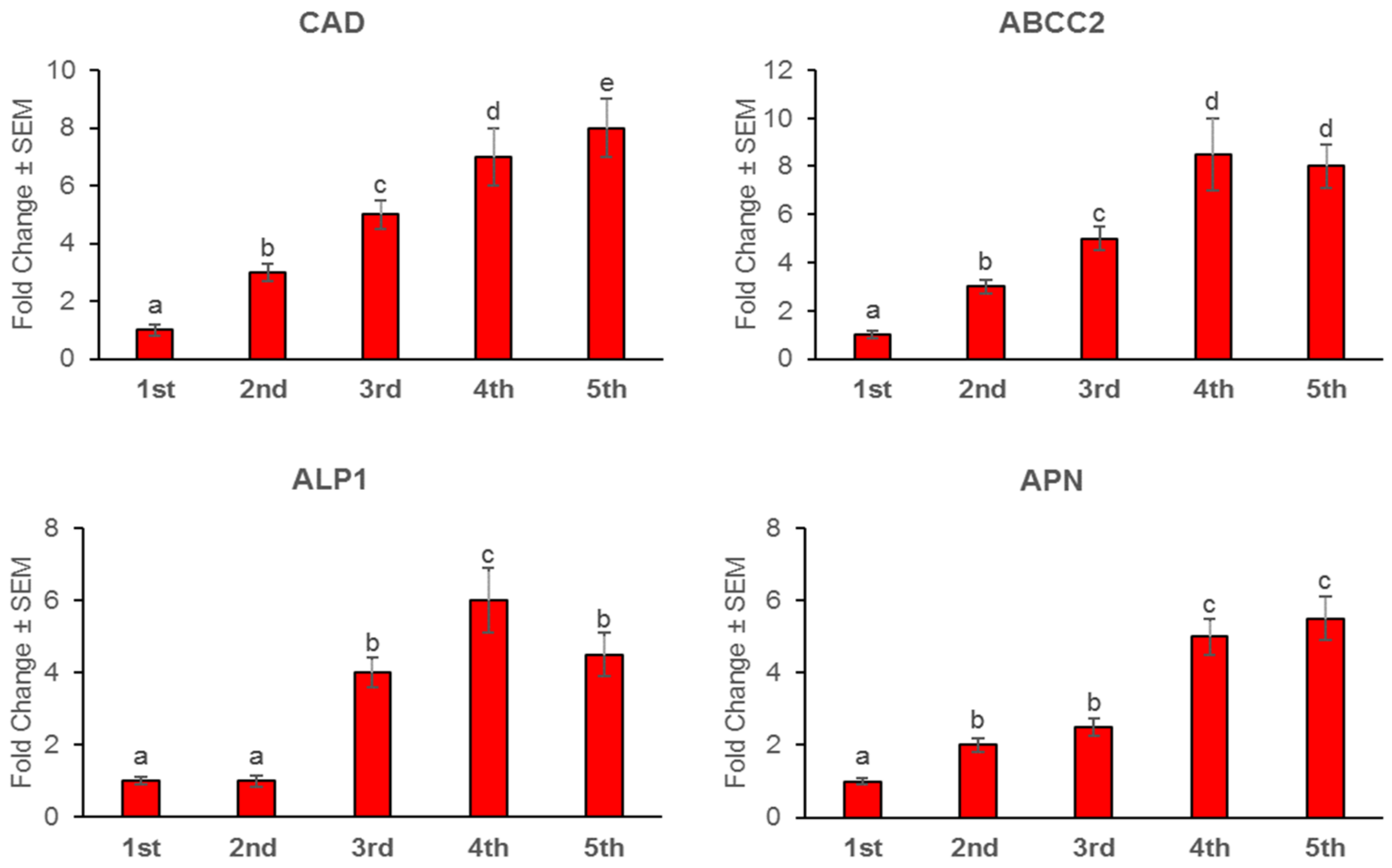

Supplement: Supplemental Information 5 — Fold change in expression of a candidate gene in different developmental (second-, third-, fourth- and fifth-instar) stages was quantified in relation to the gene’s expression in first-instar stage (value set at 1). Significant differential expression is indicated by different letters (P < 0.01, Tukey’s HSD test). S. litura GAPDH gene was used as the internal reference. Each bar represents the mean fold change value ± SE of qPCR runs in five biological and three technical replicates. [file peerj-11-14716-s005.pdf]
