# Supplementary material for: RNAi-based knockdown of candidate gut receptor genes altered the susceptibility of Spodoptera frugiperda and S. litura larvae to a chimeric toxin Cry1AcF
Source: PeerJ. 2023 Jan 24;11:e14716. doi: 10.7717/peerj.14716 (PMC9881468; doi:10.7717/peerj.14716)

Supplementary Figure 5. RT-qPCR-based detection of transcripts corresponding to CAD, ALP1, APN and ABCC2 receptor encoding genes in *S. frugiperda* fourth-instar larvae silenced (si) with different target genes. Larvae were orally ingested with dsRNA-expressing *E. coli* HT115 cells and inoculated for 24 h. Larvae force fed with GFP dsRNA and PBS were used as the non-native and negative control, respectively. Gene expression was normalized using *rps3* gene of *S. frugiperda*. Each bar represents the mean fold change value with standard error of RT-qPCR runs in five biological and three technical replicates. Treatments with different letters are significantly different at $P < 0.01$, Tukey's HSD test.

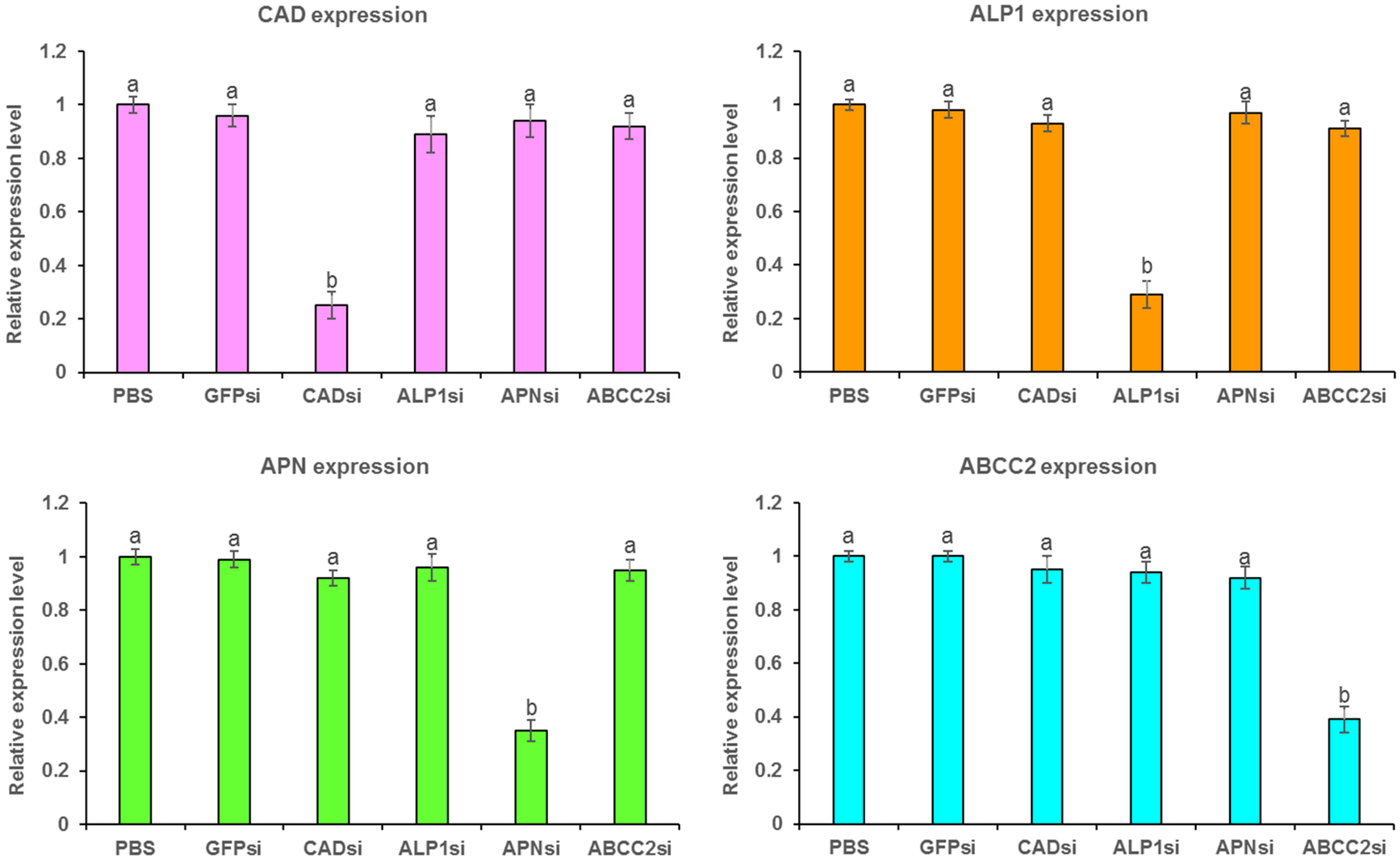

Supplement: Supplemental Information 7 — Larvae were orally ingested with dsRNA-expressing E. coli HT115 cells and inoculated for 24 h. Larvae force fed with GFP dsRNA and PBS were used as the non-native and negative control, respectively. Gene expression was normalized using rps3 gene of S. frugiperda. Each bar represents the mean fold change value with standard error of RT-qPCR runs in five biological and three technical replicates. Treatments with different letters are significantly different at P < 0.01, Tukey’s HSD test. [file peerj-11-14716-s007.pdf]
