# Supplementary material for: RNAi-based knockdown of candidate gut receptor genes altered the susceptibility of Spodoptera frugiperda and S. litura larvae to a chimeric toxin Cry1AcF
Source: PeerJ. 2023 Jan 24;11:e14716. doi: 10.7717/peerj.14716 (PMC9881468; doi:10.7717/peerj.14716)

Supplementary Figure 7. Percent pupation and adult emergence data of dsRNA-fed *S. frugiperda* **(A)** and *S. litura* **(B)** insects during 7-10 days after inoculation. Each bar with identical letter indicates no significant difference between treatments ($P > 0.01$, $n = 30$).

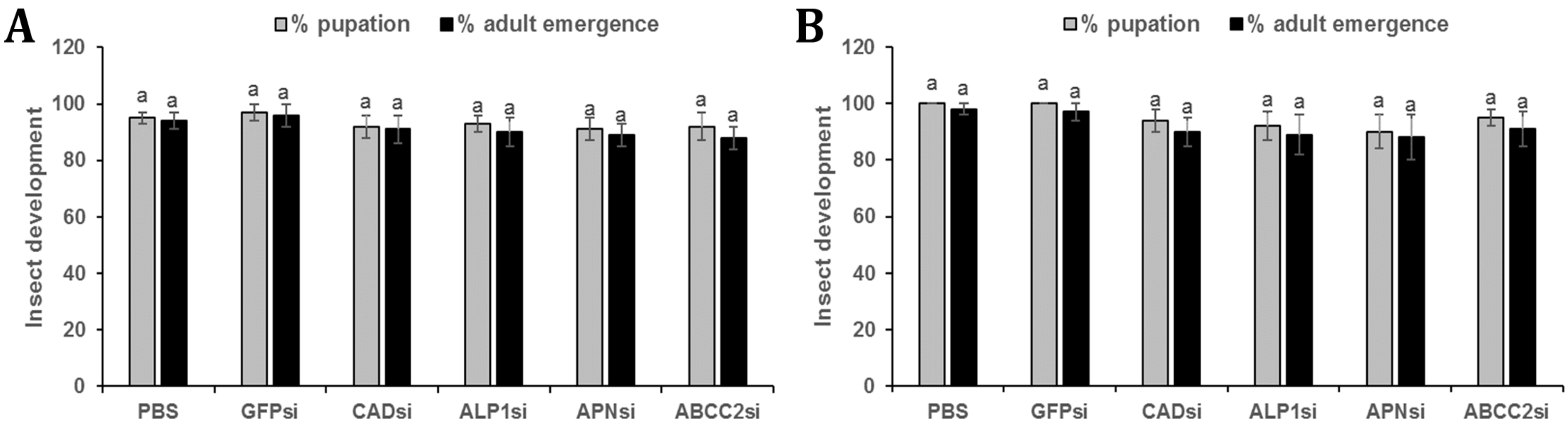

Supplement: Supplemental Information 9 — Each bar with identical letter indicates no significant difference between treatments (P > 0.01, n = 30). [file peerj-11-14716-s009.pdf]
