# Supplementary material for: RNAi-based knockdown of candidate gut receptor genes altered the susceptibility of Spodoptera frugiperda and S. litura larvae to a chimeric toxin Cry1AcF
Source: PeerJ. 2023 Jan 24;11:e14716. doi: 10.7717/peerj.14716 (PMC9881468; doi:10.7717/peerj.14716)
Supplement: Supplemental Information 11 [file peerj-11-14716-s011.docx]

>AX147205 (CAD)

GACATTCTGTGGTGAAAACATTTTTTATTTATTTTTTTCTAGTGGTTTGTGGGTACAGTGTAAACATTTT

GGAATATTGTTAAAGATTTCGGAATATTGTTAAAGTATTGACAGATAAAGCTGTAACATCACTAGAGAAG

TGAGAACTGCAAGATCATGAGATGGCGGTCGATGTGCGAATACTGACAGCAACATTGCTGGTACTCACCA

CTGCTACAGCACAGCGAGATCGATGTGGCTACATGGTAGAAATACCCAGACCAGACAGGCCTGACTTCCC

ACCTCAAAATTTTGACGGTTTAACATGGGCTCAGCAGCCACTATTACCAGCTGAGGATCGAGAAGAGGTC

TGCCTCAATGACTATGAACCTGATCCCTGGAGCAACAACCATGGTGACCAGAGAATTTACATGGAGGAGG

AGATCGAAGGTCCCGTAGTCATTGCGAAAATTAACTACCAAGGAAACACCCCTCCTCAAATAAGATTACC

TTTTCGTGTTGGTGCAGCCCACATGCTTGGAGCAGAAATTCGTGAATATCCTGACGCAACTGGAGACTGG

TATCTTGTAATTACTCAAAGGCAGGACTATGAAACTCCTGATATGCAGAGATACACGTTCGATGTGAGTG

TGGAAGGCCAGTCGCTGGTTGTAACGGTGAGGCTGGATATTGTGAACATCGACGACAATGCGCCCATCAT

TGAGATGTTAGAGCCTTGCAACTTACCGGAACTTGTTGAACCCCATGTTACAGAATGTAAATATATCGTG

TCCGACGCAGACGGTCTGATCAGTACAAGTGTTATGAGTTATCATATAGACAGCGAGAGAGGAGACGAAA

AAGTATTCGAACTGATCAGAAAAGATTATCCGGGCGATTGGACGAAGGTGTATATGGTTCTTGAATTGAA

AAAATCTCTTGATTACGAAGAGAATCCTCTACACATATTCAGAGTCACGGCTTCTGATTCCTTACCAAAC

AATAGGACCGTGGTCATGATGGTTGAAGTAGAGAACGTGGAACATAGAAATCCTCGGTGGATGGAGATCT

TTGCTGTGCAACAGTTTGATGAAAAACAGGCGAAATCGTTCACAGTGCGAGCTATTGATGGCGACACGGG

AATCAATAAACCTATATTCTATCGTATAGAAACTGAAGATGAAGACAAAGAGTTCTTCAGCATTGAGAAC

ATAGGGGAAGGCAGAGACGGTGCCAGATTCCACGTGGCTCCTATAGACAGAGACTACCTGAAAAGGGATA

TGTTTCATATAAGAATAATTGCATATAAACAAGGTGATAATGACAAAGAAGGTGAATCATCGTTCGAGAC

CTCAGCAAATGTGACGATTATAATTAACGATATAAATGATCAGAGGCCAGAACCCTTCCATAAAGAATAC

ACGATCTCCATAATGGAAGAAACTGCGATGACCTTAGATTTGCAAGAGTTTGGTTTCCATGACCGTGACA

TTGGTCCCCACGCTCAGTACGACGTTCACTTAGAGAGTATACAGCCAGAGGGGGCCCATACCGCTTTCTA

CATCGCCCCTGAAGAAGGTTACCAGGCCCAGTCTTTCACCATAGGTACTAGAATCCATAACATGTTGGAT

TATGAAGATGACGACTACAGACCAGGAATAAAGCTAAAGGCAGTAGCAATTGACAGACACGATAACAATC

ACATTGGGGAAGCAATTATTAACATTAACCTTATCAATTGGAATGATGAGCTACCTATATTCGACGAGGA

CGCCTACAACGTGACATTTGAGGAGACGGTCGGTGATGGCTTCCACATTGGTAAATACCGGGCTAAAGAC

AGAGACATCGGTGACATAGTCGAGCACTCGATATTGGGCAACGCTGCAAACTTCCTGAGAATTGACATAG

ATACTGGAGATGTGTACGTGTCACGGGACGATTACTTTGATTATCAAAGACAGAACGAAATCATAGTTCA

GATTCTGGCTGTTGATACACTAGGTTTACCTCAGAACAGGGCTACCACACAGCTCACGATATTTTTGGAA

GACATCAACAACACGCCACCTATACTGCGACTGCCACGTTCCAGTCCAAGTGTAGAAGAGAACGTTGAAG

TCGGGCACCCGATTACCGAGGGGCTAACGGCGACAGACCCAGACACCACAGCCGATTTACACTTCGAGAT

CGATTGGGACAATTCTTACGCTACGAAGCAGGGCACCAATGGACCCAACACTGCAGACTACCACGGATGC

GTAGAAATCCTGACGGTATACCCAGATCCTGACAATCACGGGAGAGCTGAGGGTCACTTGGTGGCACGTG

AGGTCAGTGATGGCGTGACCATCGATTACGAGAAGTTTGAGGTGCTGTACCTCGTCGTCAGGGTGATAGA

TCGCAACACTGTCATTGGCCCTGATTATGACGAAGCAATGCTGACGGTGACGATAATCGATATGAACGAC

AACTGGCCGATATGGGCCGACAACACGCTGCAGCAGACACTGCGCGTGCGCGAGATGGCCGACGAAGGAG

TCATCGTCGGTACACTGCTCGCCACCGACTTGGATGGCCCTCTCTACAACCGAGTCCGCTACACCATGGT

CCCCATCAAGGACACTCCTGATGACCTAATAGCGATCAACTACGTCACCGGTCAGCTGACTGTGAACAAG

GGGCAAGCAATTGACGCAGATGATCCACCTCGCTTCTACCTGTATTACAAGGTCACTGCCAGCGATAAGT

GCTCTCTTGACGAGTTCTTCCCTGTGTGCCCACCTGACCCCACTTACTGGAATACCGAGGGAGAGATAGC

GATCGCGATAACCGATACGAACAACAAAATTCCACGCGCGGAAACAGATATGTTCCCTAGTGAAAAGCGC

ATCTATGAGAACACACCCAATGGTACCAAGATCACGACGATCATCGCTAGTGACCAGGACAGAGATCGAC

CAAATAACGCGCTGACGTACAGAATCAACTACGCATTCAACCACAGGCTGGAGAACTTCTTCGCAGTGGA

CCCTGATACTGGTGAACTGTTTGTCCACTTCACCACTAGCGAAGTGTTGGACAGAGACGGAGAGGAACCG

GAGCATAGGATCATCTTCACCATCGTCGATAACTTGGAAGGCGCTGGAGATGGCAATCAGAACACAATCT

CCACGGAGGTGCGTGTTATACTGCTTGATATAAACGACAATAAGCCGGAACTACCAATTCCTGATGGCGA

ATTTTGGACCGTTTCCGAAGGTGAAGTGGAGGGAAAACGCATTCCACCAGAGATTCACGCACACGACAGA

GATGAACCATTCAACGACAACTCTCGCGTGGGATATGAAATTCGATCGATCAAATTGATCAATAGAGACA

TCGAGCTTCCTCAAGATCCATTCAAAATAATAACGATTGATGATCTCGATACCTGGAAATTCGTTGGAGA

GTTGGAGACTACCATGGACCTTAGAGGATACTGGGGAACCTATGATGTCGAGATACGTGCGTTTGACCAC

GGTTTCCCGATGCTGGATTCATTCGAGACCTACCAACTAACCGTCAGGCCATACAACTTCCATTCACCGG

TGTTTGTGTTCCCAACTCCTGGCTCAACCATCAGGCTTTCTAGGGAGCGTGCTATAGTCAATGGTATGCT

GGCTCTGGCTAATATCGCGAGCGGAGAGTTCCTCGACAGACTCTCTGCCACTGATGAAGATGGGCTACAC

GCAGGCAGAGTAACTTTCTCCATAGCTGGAAACGATGAAGCTGCGGAATATTTCAATGTGTTGAACGACG

GTGACAACTCAGCAATGCTCACGCTGAAGCAAGCATTGCCCGCTGGCGTCCAGCAGTTTGAGTTGGTTAT

TCGGGCCACGGACGGCGGGACGGAGCCGGGACCTAGGAGTACCGACTGCTCCGTCACTGTGGTGTTTGTG

ATGACGCAGGGAGACCCCGTGTTCGACGACAACGCAGCTTCTGTCCGCTTCGTTGAAAAGGAAGCTGGTA

TGTCGGAAAAGTTTCAGCTGCCTCAGGCCGATGACCCCAAAAACTACAGGTGTATGGACGACTGCCATAC

CATCTACTACTCTATCGTTGATGGCAACGATGGTGACCACTTCGCCGTGGAGCCGGAGACTAACGTGATC

TATTTGCTGAAGCCGCTGGACCGCAGCCAACAGGAGCAGTACAGGGTCGTGGTGGCGGCTTCCAACACGC

CTGGCGGCACCTCCACCTTGTCCTCCTCACTCCTCACCGTCACCATCGGCGTTCGAGAAGCAAACCCTAG

ACCGATCTTCGAAAGTGAATTTTACACAGCTGGCGTCTTACACACCGATAGCATACACAAGGAGCTCGTT

TACCTGGCGGCAAAACATTCAGAAGGGCTTCCTATCGTCTACTCGATAGATCAAGAAACCATGAAAATAG

ACGAGTCGTTGCAAACAGTTGTGGAGGACGCCTTCGACATTAACTCTGCAACCGGAGTCATATCGCTGAA

CTTCCAGCCAACATCTGTCATGCACGGCAGTTTCGACTTCGAGGTGGTGGCTAGTGACACGCGTGGAGCG

AGTGATCGAGCAAAAGTGTCAATTTACATGATATCGACTCGCGTTAGAGTAGCCTTCCTGTTCTACAACA

CGGAAGCTGAAGTTAACGAGAGAAGAAATTTCATTGCACAAACGTTCGCCAACGCGTTTGGTATGACATG

TAACATAGACAGCGTGCTGCCGGCTACCGACGCCAACGGCGTGATTCGCGAGGGGTACACAGAACTCCAG

GCTCACTTCATACGAGACGACCAGCCGGTGCCAGCCGACTATATTGAGGGATTATTTACGGAACTCAATA

CATTGCGTGACATCAGAGAGGTACTGAGTACTCAGCAATTGACGCTACTGGACTTTGCGGCGGGAGGGTC

GGCAGTGCTGCCCGGCGGAGAGTACGCGCTAGCGGTGTACATCCTCGCCGGCATCGCAGCGTTACTCGCC

GTCATCTGTCTCGCTCTCCTCATCGCTTTCTTCATTAGGAACCGAACACTGAACCGGCGCATCGAAGCCC

TCACAATCAAAGATGTTCCTACGGACATCGAGCCAAACCACGCGTCAGTAGCAGTGCTAAACATTAACAA

GCACACAGAACCTGGTTCCAATCCCTTCTATAACCCGGATGTTAAGACACCTAACTTCGACACTATAAGC

GAAGTATCCGATGACCTGCTTGATGTCGAAGACTTGGAACAGTTTGGAAAGGATTACTTCCCACCCGAAA

ACGAAATTGAGAGCCTGAATTTTGCACGTAACCCCATAGCGACACACGGGAACAACTTTGGCGTAAACTC

AAGCCCCTCCAACCCAGAGTTCTCCAACTCCCAGTTTAGAAGTTAAACTAAATACACTTTTATCACTTGC

ATAGACTTATGTATTTAATAATTTTACATTTTTTACATTAAATATAAATGTTTTATATGTAATAATAGTG

TGATAAAATGTACGTAACAATCAACATAGCTGTTGTAGGTTCGTAAATAACATACTCGTAATGTATAAGT

GTTATGTTTATATATAGAAATAAAAATATTAAATATTAAAAAAAAAAAAAAAAAAAAAAAAA

FP:GTGTTGAACGACGGTGACAA

RP:GACCCTGTACTGCTCCTGTT

411 bp (3768-4178)

GAGAGCTGAGGGTCACTTGG

ATATCGGCCAGTTGTCGTTC

182 bp (2282-2463)

>OL955491 (ABCC2)

ATGATGGACAAGTCCAACAAGAACACCGCTGCTAACGGCAACGGTGGTCAACGTGCTGGCGAGCCTAAAG

AGCGTGTCCGCAAGAAGCCCAACATCCTGTCCAGGATCTTCGTGTGGTGGATCTTCCCTGTGCTGATCAC

CGGCAACAAGCGTGACGTGGAAGAGGACGACCTGATCGTGCCCTCCAAGAAGTTCAACTCTGAGCGTCAG

GGCGAGTACTTCGAGCGCTATTGGTTCGAGGAAGTGGCTATCGCTGAGCGCGAGGACCGTGATCCATCTC

TGTGGAAAGCTATGCGTCGTGCTTACTGGCTGCAGTACATGCCCGGTGCTATCTTCGTGCTGCTGATCTC

TGGACTGCGTACCGCTCAGCCTCTGCTGTTCTCCCAGCTGCTGTCTTATTGGAGCGTGGACTCCGAGATG

TCCCAGCAGGATGCTGGACTGTACGCTCTGGCTATGCTGGGTATCAACTTCATCACCATGATGTGCACCC

ACCACAACAACCTGTTCGTGATGCGCTTCTCCATGAAGGTCAAAATCGCTGCTTCTTCCCTGCTGTTCCG

CAAGCTGTTGCGTATGTCCCAGGTGTCCGTTGGAGATGTGGCTGGTGGCAAGCTGGTCAACCTGTTGTCC

AACGATGTGGCTCGTTTCGACTACGCTTTCATGTTCCTGCACTACCTGTGGGTCGTGCCCCTGCAAGTTG

GTGTCGTGCTGTACTTCGTGTACGACGCTGCTGGATGGGCTCCTTACGTGGGACTGTTCGGTGTCATCAT

CCTGATCATGCCACTGCAGGCTGGCCTGACCAAGCTGACTGGTGTTGTGCGTCGTATGACCGCTAAGCGT

ACCGACAAGCGTATCAAGCTGATGTCCGAGATCATCAACGGCATCCAAGTGATCAAGATGTACGCTTGGG

AGAAGCCCTTCCAGCTGGTGGTTAAGGCTGCTCGCGCTTACGAGATGTCTGCTCTGCGCAAGTCCATCTT

CATCCGTTCCATGTTCCTCGGCTTCATGCTGTTCACCGAGCGTTCCGTGATGTTCCTGACCGTGCTGACT

CTGGCTCTGACCGGAAACATGATCTCTGCTACCCTGATCTACCCGATCCAGCAGTACTTCGGCATCATCA

CTATGAACGTGACCCTGATCCTGCCTATGGCTTTCGCTTCCTTCAGCGAGATGCTCATCTCCCTCGAGCG

TATCCAGGGTTTCCTGCTGCTGGACGAGCGTTCTGACATCCAGATCACCCCTAAGGTCGTGAACGGTGCT

GGTTCCAAGCTGTTCAACAACTCCAAGAAAGAAGGTGGCCTGGAAACCGGCATCGTGCTGCCTACCAAGT

ACTCCCCTACCGAGGCTAACATGGCTCGTCCCATGCAGGACGAACCCAACATGGCTGACTACCCCGTGCA

GCTGAACAAAGTGAACGCTACCTGGGCTGACCTGAACGACAACAAAGAGATGACCCTGAAGAACATCTCC

CTGCGCGTGCGCAAGAACAAGCTGTGCGCTGTTATCGGTCCTGTCGGTTCCGGAAAGACCTCTCTGCTGC

AACTGCTGCTGCGTGAACTGCCTGTGACCTCCGGAAACCTGTCCATCTCCGGTACTGTGTCCTACGCTTC

CCAAGAGCCTTGGCTGTTCCCTGCTACCGTGCGTGAAAACATCCTCTTCGGCCTCGAGTACAACGTGGCC

AAGTACAAAGAAGTGTGCAAAGTCTGCTCCCTGCTGCCTGACTTCAAGCAGTTCCCTTACGGCGACCTGT

CTCTCGTGGGAGAACGTGGTGTTTCCCTGTCTGGTGGACAGCGTGCTCGTATCAACCTGGCTCGTGCTGT

GTACCGCGAGGCTGACATCTACCTGTTGGACGACCCTCTGTCCGCTGTGGACGCTAACGTTGGTCGTCAA

CTGTTCGACGGTTGCATCAAGGGTTACCTGTCCGGCAAGACCTGCATCCTGGTCACCCACCAGATCCACT

ACCTGAAGGCCGCTGACTTCATCGTGGTGCTGAACGAGGGTTCCGTCGAGAACATGGGATCTTACGACGA

GCTGATGAAGACCGGCACCGAGTTCTCTATGCTGCTGTCCGACCAGGCTTCCGAAGGTTCCGACACCGAT

AAGAAAGAAAGGCCGGCCATGATGCGCGGTATCTCCAAGATGTCCGTGAAGTCCGACGACGAAGAGGGCG

AAGAGAAGGTGCAAGTGCTGGAAGCTGAGGAACGCCAGTCCGGTTCTTTGAAGTGGGACGTGCTGGGCCG

TTATATGAAGTCCGTGAACTCCTGGTGTATGGTCGTGATGGCTTTCCTGGTCCTGGTCATCACCCAGGGT

GCTGCTACCACTACCGACTACTGGCTGTCCTTCTGGACCAACCAGGTGGACGGTTACATCCAGACTCTGC

CTGAGGGCGAGTCCCCTAATCCTGAGCTGAACACCCAAGTCGGACTGCTGACCACCGGTCAGTACTTGAT

CGTGCACGGTTCTGTGGTGCTGGCTATCATCATCTTGACCCAAGTCCGTATCCTGTCCTTCGTGGTCATG

ACTATGCGCGCTTCCGAGAACCTGCACAACACTATCTACGAGAAGCTGATCGTGGCCGTCATGCGATTCT

TCGACACTAACCCTTCCGGTCGCGTGCTGAACCGTTTCTCCAAGGACATGGGTGCTATGGACGAGCTGCT

GCCTCGTTCCATGCTCGAGACTGTGCAGATGTACCTGAGCCTGGCTTCCGTGCTGGTCCTGAACGCTATC

GCTCTGCCTTGGACTCTGATCCCCACCACCGTCCTGATGTTCATCTTCGTTTTCTTGCTGAAGTGGTACA

TCAACGCTGCTCAGGCTGTGAAGCGTCTGGAAGGTACTACTAAGTCCCCTGTGTTCGGAATGATCAACTC

CACCATCTCTGGCCTGTCTACCATCCGCTCCTCTAACTCCCAAGACCGTCTGCTGAACTCCTTCGACGAC

GCCCAGAACTTGCACACCTCCGCTTTCTACACCTTCCTCGGTGGTTCCACCGCTTTCGGACTGTACCTGG

ACACCCTGTGCCTCATCTACCTCGGTATCATCATGTCGATCTTCATCTTGGGCGACTTCGGCGAGCTGAT

CCCTGTGGGTTCTGTCGGACTGGCTGTGTCCCAGTCTATGGTGCTGACTATGATGCTGCAGATGGCTGCT

AAGTTCACCGCTGATTTCTTGGGCCAGATGACCGCTGTCGAGCGTGTGTTGGAGTACACCAAGCTGCCCA

CTGAGGAAAACATGGAAACTGGTCCTACCACTCCTCCTAAAGGATGGCCTTCTGCTGGCGAAGTGACCTT

CTCCAACGTGTACCTGAAGTACTCTCCAGACGACCCACCTGTCCTGAAGGACCTGAACTTCGCTATCAAG

TCCGGCTGGAAAGTGGGAGTCGTTGGACGTACTGGTGCTGGCAAGTCATCTCTGATCTCCGCTCTGTTCC

GTCTGTCCGACATCACCGGCTCCATCAAGATCGACGGCCTGGACACTCAGGGTATCGCTAAGAAGCTGCT

GCGCTCTAAGATCAGCATCATCCCTCAAGAGCCCGTGCTGTTCAGCGCTTCCCTGCGTTACAACTTGGAC

CCCTTCGACAACTACAACGACGAGGACATCTGGCGTGCTCTCGAGCAGGTCGAGCTGAAAGAGTCTATCC

CCGCTCTGGACTACAAGGTGTCCGAAGGTGGCACCAACTTCAGCATGGGACAGCGTCAACTCGTGTGCCT

GGCTAGGGCTATCCTGCGTTCTAACAAGATCCTCATCATGGACGAGGCTACCGCTAACGTGGACCCTCAA

ACCGACGCTCTGATCCAAAAGACCATCAGAAAGCAGTTCGCTACCTGCACCGTGCTCACTATCGCTCACC

GTCTGAACACTATCATGGACAGCGACCGTGTGCTGGTCATGGACCAAGGTGTCGCTGCTGAGTTCGATCA

CCCTTACATCCTGTTGAGCAACCCCAACTCTAAGTTCTCCAGCATGGTCAAAGAAACCGGCGACAACATG

TCCCGCATCCTGTTCGAGGTGGCCAAGACTAAGTACGAGTCCGACTCCAAGACCGCCGATTACAAGGACC

ACGACGGCGACTACAAAGATCACGACATCGACTATAAGGACGACGACGACAAGTAA

TGCTGGCAAGTCATCTCTGA

CTTTTGGATCAGAGCGTCGG

406 bp (3396-3801)

GTCAGGGCGAGTACTTCGAG

CACGCTCCAATAAGACAGCA

203 bp (206-408)

>MT506048 (ALP1) ATGAGGTCGCTACTGACTTACCTAGTGGCCGCCGTGATGGTGGCGGCATGTGTCCGCGGGGACCGGTACC

ACCCCGCGGACCCCGGCAGCAGAGCTGACACCGTTGTGAACCGTGCCGAGACCTCAGCCAACTACTGGGC

CCAAGAAGCGCAGGCTGCAATCAATGCCCGGCTGGCGCACAAGGAGAGCGTGAAGAAGGCGCGCAACGTG

GTCATGTTCCTGGGCGACGGCATGTCCGTGCCCACGCTCGCCGCCGCGCGGACGCTGCTCGGCCAGCGCC

GCGGGCACACCGGCGAGGAGGATAAACTGCATTTTGAAACATTCCCCACCGTTGGATTGACTAAGACGTA

TTGCGTGAACGCTCAGATCCCAGACTCCGCGTGCACTGCTACTGCGTACTTATGCGGTGTCAAAACAACT

TACGGAGCTATTGGAGTGAATGCGGAGGTGCCACGGAAAGGCTGCGAGGCGTCCACCGACACCAGCCGAC

ACGTGGAGTCCATCGCCGAGTGGGCGCTGGCCGACGGCCGCGACGCTGGTATCGTGACGACGACGCGCAT

CACCCACGCGTCGCCGGCCGGCGTGTACGCCAAGGTGGCGGACCGCAACTGGGAGCACAACCAGGCGGTG

GAGAACGATGGCTTCGACACGGACAAGTGCCCGGATATCGCACTGCAGCTCGTGCATAAGCACCCCGGGA

ATAAACTCAAGGTTATTTTAGGCGGAGGAAGACTAAACTTTTTGCCAAATGATGTGAAAGACGAAGAAGG

AGTATATGGAAACCGAACAGACACCCGCAACCTCATCGAAGAATGGGCACAAGACAAGGAAGATCGTAAA

GTTACTCATAAATATGTTTGGAATCGTGAGCAGCTGATGAGTCTTAAAGATGATCTTCCTGAGTACCTTT

TAGGACTTTTCGAAAGTAATCATCTTCAGTACAACATGCAGGCAGATCCTAATACTGAGCCCACGTTGAC

TGAGCTAACTGAGATAGCAATCAAGTCGCTAAGTAGAAACGAGAAAGGTTTTTTCCTGTTCGTGGAAGGC

GGTCGTATCGACCACGCACACCATCGCAACTGGGTAGAGCTAGCGCTGGACGAGACGCTGGAGATGGACA

AGGCCGTCGCGCGCGCCGCCGAGCTGCTCTCCGAGGACGACTCGCTCATTGTGGTCACAGCAGACCACTC

CCACGTCATGGCTTACAATGGCTACTCGGCCCGTGGACATGACATCCTCGGCCCTTCCAGAGACTTGGAC

CTGGACGGAGTGCCTTACATGACGCTGTCGTACACCAACGGGCCCGGCTTCCGTTCGCATATGAACGGTA

TACGCCCCGATGTCACCGCTGAAGACGATTTCAGAAAAGACGGATGGTTGGCTCACGTAGATGTTCCGCT

GATAGACGAGACGCACGGTGGGGACGACGTGGCGGTGTTCGCGCGCGGGCCGCACCACTCCATGTTCACG

GGGCTGTACGAGCAGAGCCAGCTGCCGCACCTCATGGCGTACGCCGCCTGCATCGGCCCCGGCAGACACG

CCTGCAGCGGCGCCGCGCATGCGCTGGCCCAGCCTGTGCTGCTGCTCTCTCTCCTTGTACTGCTCACTTC

ACTATTCCAACAATGA

GACTCGCTCATTGTGGTCAC

ACAAGGAGAGAGAGCAGCAG

440 bp (1159-1598)

CAAGGAGAGCGTGAAGAAGG

TCTGAGCGTTCACGCAATAC

188 bp (180-367)

>MT673677 (APN)

ATGGCGAATCGCTGGTTTAGCCTCTTATTGGGGGCCATTTTACTCCAGTCTGTGCTGGCGTTTGGCCCAA

TCGATGTCACGGATGCCGAATGGATTGAATACATGGGGCTGATTAATAACCCCAATTACCGGTTGCCAAC

TACCACTAGACCAACTCATTACAAAGTTAGACTGCAACCAAATTTAGACCAAAATTTTGAGCTTAACGGT

GATGTCGAAATAAACATTAAAGTTGAAAGTGAGAATCAACCAATCAACGAAATCAAACTTCACTGCCAGG

ATATGGTAATCAACAGTTTGACCGTAACCTCAACTACAAATACCCAAGTAAACCTTGCTCAGGGCACTCA

GTTTGTATGCGAAGAAACTACTTCCTTTTTAACGATTCCAACCACCACTCAGCTACCTAATGGAAATGAG

TACATCATCAAGATATCATTTGTTGGAAAGTTGCAAAGTGGTATGAGAGGTTTCTACAGGAGTTGGTTCT

TTGATGAAAATAAGCAGAAAAGATGGATGGCTACTACCCAATTCCAGCCTGGCCATGCTCGTCAAGCGTT

CCCTTGCTACGATGAACCTGGCTTCAAAGCCACATTTGATATAATATTGGTTAGAGACGATTCACTTATT

TCATTATCAAACATGCCCATAGAGGCAACTGTACCATCTACCTTATATCCCCAGAAAAAAGAGGACATAA

ACTACACTACGCCTATTATGTCCACATACTTACTGGCTTTCATAGTAGCGGATTACAAACGGATCGAGTC

TGGAACAAATGTGAATAGACCATTCCATATCTATGCTCGAGGTAACGTTGGAGATACAGGCAAATATTCT

TTGGAGGTCGGTGAAAAACTTTTGACGTTAATGGAATTGTATACTCAATACAATTACTACACAATGGCTT

CCCACATGGAAATGAAGCAAGCTGCTATTCCTGACTTTAGCGCCGGTGCTATGGAAAACTGGGGCCTCTT

AACCTACAGGGAAGCTCTCATTCTGTACGATCCTGAAAACAGCAACAATTTTTACAAACAACGCATAGCC

AACATTATATCTCACGAAATTGCACATATGTGGTTCGGTAACCTCGTCACATGCGCTTGGTGGGACACTC

TTTGGTTAAACGAAGGTTTCGCTAGATACTACCAATACTACATGACCGACAAGGCTGAGCCACACATGGG

CTTTAAGACACGTTTCATCGTAGAACAGCTGCAAGTGGCCATGCTCTCCGACTCCTTCTCAAATGCTCAC

GCACTTACTAACCCTGCAGTGTCTGATCCAGATTCTGTGAGTAACCACTTTTCAACCATCACTTATGCCA

AAGGTGCCTCTATACTTAGAATGACAGAACATCTGCTTGGTGGGGACACCTATGAGAAGGGTCTTCGGGA

ATATTTAAAGAAAAGAGAATTTAATACCGCTGAACCAAAGGATTTATTCGAAAGCTTGGACGCTGCTGCT

AATGCTGACAATTCTCTAGCTGATTACGATGATATGACAATTGCGAAGTACTTCGCATCGTGGTCTGAAA

AAGCAGGGCATCCTCTATTGACAGTTCATGTAGATCACGCTAGCGGCCGTATGACCGTCGTACAAACTCA

ATTTGATGTCAACAGTGGTGTGTCTTCGGACAATGGTTTATGGCACATTCCTTTAACTTGGACTAGGGCT

GGAAATCCTGAATTTGAAAATCTTAAGCCTTCTGAATTCATGAGTGGCCCACTAAAAATTATTAACCGAG

GAAGTACTGGTAGAGAATGGGTTATTTTCAACAAACAACAATCTGGTTTCTACAGAGTAAACTACGACGC

CACTACGTGGGCTCTTCTTACTCAAGCTTTGCGAAGTAACCAGAGGGAGGCCATCCACGAATACAACCGC

GCTCAGATTGTGGATGACGTGTTCGTGTTAGCTAGATCTAACATCTTGTCCTATACGCGAGCGTTGAACA

TTCTTTCCTTCCTTGAATTTGAAGACAAGTACGCTCCTTGGGTAGCTGCTATTACTGGATTCAACTTTGC

GCTTCGGAGATTGGCTCATAAGACTGAAGAACAACAAAAACTGAAGGATATAATCTTTAAATCGAGTGCG

GCGATCATCCAGCGTCTCGGTTACACCGAGGCGTCCAACGCCGATCCTCTTTTCATGGACAACTTGCTCC

GTATGCATCTCATGACCTTCCTTTGTAACGCTGGACATGCGCAATGCTCCCAAACAGGAAGAGAATATTT

CAAGGCATGGAGAGAGAGTGGGACAAGAATTCCGCCAAACATGCGTCCTTGGGTATATTGCGAAGGTCTT

CGCACTGGAGATTTAGCTGATTTCGATTATTTCTGGGACCGTTATGTGGATGAAGATTTATCTAACGAGA

AAGTCGTGATGATCGGTGCAGCTGGTTGCACAGGAAATACAGCGGCCTTACACAAATTCCTCAGTGTAAT

CGTTGACCCAAAACCGACTGAACAATCAACTGAACTAATCAGACCTCAGGACTACAGCGCTGCTATTAGT

TCTGCTGTTACAAGCAACGAATACAATACTATGAAAGTACTTGAGTGGCTTAAGGATAATCCTTCACATC

TTCAGAATGGAAATGGCGTAAGTCTTTTACGATCTGCAGCAAGCCGATTATTGAACGAGTTGGATATTCT

TCAGGTTGAAAACTGGTTTATTACTATTACCTCAGATGAAGCTATACAAGCGATTAAGGATGGCTTTGCT

ACATCAAGGAGTAACATAAAATGGTATGAGAGTAGGGTAGGGGAGTTCAGTGATTACTTCGAAACAGGAT

ATTTTGACGATTTGACTGGCGGATCAGAAACACCTGATCCTACTACCCCTGAACCTACTACCGCTGAACC

TACTACGCCTGAACCAACTACCCCTGAACCTACTACCGCTGAACCTACTACCGATGAATCTACTCCCGCT

GAACCTACTACCGCTGAACCTACTACCGCTGAACCCGAACCTGGCTCTGCGAATATCGCTTCTCTCAGTT

TCTTCACTTTGCTAGTCACACTCATCATCAACATGGTTAA

CTTGCTACGATGAACCTGGC

CCAGTTTTCCATAGCACCGG

410 bp (563-972)

CTATACGCGAGCGTTGAACA

TGAGATGCATACGGAGCAAG

242 bp (1941-2182)
