# Supplementary material for: RNAi-based knockdown of candidate gut receptor genes altered the susceptibility of Spodoptera frugiperda and S. litura larvae to a chimeric toxin Cry1AcF
Source: PeerJ. 2023 Jan 24;11:e14716. doi: 10.7717/peerj.14716 (PMC9881468; doi:10.7717/peerj.14716)
Supplement: Supplemental Information 12 [file peerj-11-14716-s012.docx]

>JN687590 (CAD)

ATGGCGCTTGATGTGCGATTACTGATAGCAACATTGCTGATACTCACCACTGCCGCAACCGCTCAACAAG

ATCGATGTGGCTACATGGTGTTAATACCCAGACCAGAGAGGCCTGACTTCCCACCACAAAATTTTGACGG

TTTGACATGGGCTCAGTGGCCACTGTTACCAGCTGAGGATCGAGAAGACGTCTGCATCAATGACTATACA

CCTGATCCCTTTAGCACCAACCATGGTTACCAGAAAATTTACATGGAGGAGGAGATCGTAGATGACGTGG

CCATTGCAAAGCTTAATTACCGAGGTAACGGCAGACCTCAAATACAAACACCTTTTATCATGGGAGCAGC

CCACATGCTTGGAGCAGAAATTCGTAGATACCCTGACGAAAATGGAGACTGGCATCTTGTTATCACTCAG

AGGCAGGATTATGAAACTCCTGGTATGCAAGGATATATATTCAACGTGGGAGTGGAGGGCGAGTCGCTGG

TTGTGATGGTGACCCTAGACATTGTGAACATCGACGACAATACTCCAATCATTGAGATGCTAGAGCCTTG

CAGCATACCGGAACTTGGTGAACCCGGTATTACAGAATGTAAATATGTCGTGACCGACGCAGATGGTGAG

ATCAGTACAAGTGTTATGAGATTTGAAATAGACAGCGAGAGAGGAGACGAACTAGTATTCGAACTGACCA

GAGAAAATATCCCGGGCGATTGGTTCTCGGTGTATATGGTCCTTGAAGTGAAACAGCCTCTTAATTATGT

TGAGAATCCTCTACACATATTCAGAGTTACGGCTTTGGACTCGTTACCAAACCCACGTACAGTCGTCATG

ATGGTTGAAGTAGAGAATGTGGAACACAGAAACCCTCAATGGATAACGATCTTTGCTGTGCAACAATTTG

ATGAGAAACTGGAGAGATCGTTCCCAGTTAGAGCTATAGATGGCGACACGGGAATCAATAAACCTATATT

TTATCGTATAGAAACTGAAGAAAGAGACAAAGAATTCTTCAGCATTGAAACTTTAGGAGAAGGCAGAGAT

AGTGCCATGTTCCACGTGGCAGCTATAGACAGGGACACACTAAAAAGGGATATGTTTAATGTAACAATAA

TTGCGTATAAGTATGGTGATATACCTGTATCCAGTAGTGAACAAGGACAGGCAGTTCATGATGACGAAGG

TGATAGTGACGAAGGTGGTCCACGGTTATTTGAGACCCCAACGAATGTGATGATCATAATTAACGATATA

AATGATCAGAGGCCTGAACCTTTCCAGAAGGAATACACGATCTCCATAATGGAAGAGACTGCGATGACGT

TACCTTTGACAAACAAACAAACAATCTTCGGTTTTCATGACCGTGATATTGGTCCCCACGCACAGTACAA

AGTTCACTTAGAGAGTATACATCCAGAGGGGATTCATAACGCCTTCTACATCGCCCCTGAAGAAGGTTAC

CAGGGGCAAGAGTTTATCATTGGTACAGTTGACCACCATATGTTGGATTATGATCGAGGCTATGACCCCA

CGAAAGGAATAAAGCTAAAGGCAGTAGCAATTGACAAGGATAATAACGATCACATTGGAGAAGCAATAAT

AAACATTAACCTTATCAATTGGAATGATGAACTGCCTGGGTTCGACAGAGATAGCTACAACGCAGATTTT

AAGGAAACGGTCGAAGCTGACTTTCGCATTGGTACATACCAGGCTACAGACGACGACATCGGTGACATTG

TTGAGCACACGATATTGGGCAACGCTGCCAACTTCCTGAGGATTGACCTAATTACTGGAGACGTCTACGT

GACAGTAAACGATGCCTTTGATTACCACAGACAGAACGAAATCTTTGTTCAGATTCAAGCTGTGGATACA

CTAGGTTTGCCACAAAACAGGGCTACAACACAGCTAGTCATACATTTGGAAGACGTCAACAACACGCCAC

CTACCCTGCGACTGCCACGTCAAAGTCCAAGTGTAAAAGAGAACGTTAAAGACGGATTCTTGATAACCGA

AGGGCTGACGGCGACGGATCCTGACACAACGGCCGATCTATACTTCGAGATCGATTGGAGTGCCTCATAC

GCTACGAAGCAAGGCAGGAATGCACCCCCCACTTCAGAATACCACGGATGTGTAGAAATCCTGACGGTAT

ACCCAGATCCTAACAATCGCGGGCTAGCTGAGGGACACTTGGTGGCACGTGAGGTCAGAGAGGGCGTGAC

CATCGATTACGAGGAGTTTGAGGTGCTGTACCTCGTTGTGAGGGTGATAGACCTCAACACCGCCATTGGC

GATGATTATGACGAAGCAATGATGACGGTGACAATAATCGATATGAACGACAACAGGCCGTTATGGGTGG

CGGGCACGCTGACACAACCACTGCGCGTGCGTGAGATGGCCGACGAAGGTGTCATCATCGGTACCCTGCA

GGCCACCGACATCGATGGCCCTCTCTACAACCGAGTCCGTTACACCATGATTCCCATCAATGACACTCCT

GAGGAACTGGTAAAGATCGACTACGTCACCGGTCAGCTGACTGTGAACAAGGGGCAAGCAATTGACGCAG

ATATTCCACCGCGCTTCCATCTGTATTACAAGATTACAGCCAGCGACAAGTGCTCCCTTGACGAGTTTTT

TACAGTATGCCCACCTGATCCCACCTTCTGGAATACCGACGGAGAGATAGCGATCGAGATAACTGATACG

AACAACAAAGTTCCACACGCGGAAACAGATCAGTTCCCTAAAGAAGTGCGCATCTACGAAAATGAAAGTA

ATGGTACCGTGATCACTACGATCATAGCAAGCGACTTGGACAGAGATTACCCAAACAACGAGCTGACGTA

CAGAATCAACTACGCATTCAACAATAGACTGGAGAACTTCTTCGCAGTGGATCCTAACACTGGCGTTCTG

AGAGTACACTTCGCTACTGAGGAAGTGTTGGACAGGGATGGAGATGAACCGGAGCATAGGATCATCTTCA

CCATCGTCGATAACTTGGAAGGCGCTGGAGATGGCAATCAGAACTCCATCTCCACGGAGGTGCTTGTTAT

ACTGCTTGATATAAACGACAATGCGCCGGAACTGCCAGCTTCCGTTGGCGAATTCTGGACCGTTTCCGAA

GGTGTGGTCGAGGGATACCACATTCCACCAGAGATTCATGCACACGACAGAGATGAACCATTCTCAAACA

ACTCTCGCGTCGGATATGAAATTCTATCAGTCACATTGATCAATAGAGAGATTGAACTTCCTCAAGATCC

ATTCAAAATAGAAACGATTAGTAATTTCGAAACCTGGAGATTCGTAGGAGAGTTGGTGACTACCATGGAC

CTTAGAGGATACTGGGGCACCTATGAAGTCGAAATACGTGCGTTTGACCACGGTGAGCCGCCACTGTATT

CAATCGAGACCTATGAACTAACCGTCAGGCCATACAACTTCCATTCACCGGTGTTTGTGTTCCCAACGCC

TGGCTCAACCATCAGGCTTTCCAGGGAGCGTGTCATAGTCAATGGTATGCTGGCTCTGGCTAATATCGCG

AGTGGGGAGTTCCTCGACCGACTCTCCGCTACTGATGAAGACGGGTTAGAAGCAGGCAGAGTGACTTTCT

CTATAGTTGGAAACGATGAAGCTGAGGAGTATTTTAATGTGTTGAACGATGGTGATAATTCAGCACTGCT

CACACTGAAACAAGCTTTGCCCGATGGTGTCCAACAGTTTGAGTTGGTTATTCGTGCCACGGATGGTGGG

ACGGAGCCAGCACCCAGGACCACCGACTGCCCCGTCGCTGTGCTGTTTGTGTTGACGCAGGGAGAGCCTG

TGTTCTCCGAAAACTCAGCTACTGTCCGTTTCGTAGAAAAGGAAGCTGGTATGTTGGAGAGGTTCGAGCT

GCCTCAAGCCGAGGACCCCAAGAACTACAGGTGTACGGAAGACTGCCATACCGTCTACTACACTATCATT

GACGGCAACAACGGTGACTACTTCGCTGTGGAACCGGAGACTAACGTGATCTATTTGGCGAAGGAGCTGG

ACCGCAGCGAACGGGAGCAGCATAGTATAGTGATAGCGGCTGCCAATACGATTGGCGTTACCACAGCCAT

GCCTTCCTCACAGCTCACCGTCACCATCGACGTCCGAGATGCGAACCCTAGACCTATCTTCGGCAGAGAA

CTTTACACTGCTGGCATCTTACACACAGATAACGTACACAGGGATCTCATTTACCTTACGGCAACACACA

CTGAAGGACTCCCTATCACCTACCCCATAGACCTAGAAACCATGCAAGTAGACGAGTCGTTACAAATTGT

TATGGAGGACGCCTTCAACATCAACTCTGAGACCGGGGTTATCTCGCTGAACTTCCAGCCAACACCGGCT

ATGCACGGCCACTTCGATTTCGAAGTGGTGGCTAGTGATGCAAATGAAGTGAGTGATCGAGCAAAAGTTA

TAATCTACATGATATCGACACGTGTCAGAGTAGCCTTCCTGTTCGAAAATACGGAAGCTGAAGTCAACGC

GAGAAGAGATTTCATCGCGCATACATTCTCGAACGTGTTCGATATGACGTGTAACATAGACAGCGTGCTG

CCCGCTACCGACGCCAACGGCGTGGTGCGGGAAGGTTACACAGAACTCCAGGCGCACTTCATCCGAGACG

GCATGCCGGTGCCAGCCGAGTTTATTGAAGAGTTGTTTTCGGATGTCAACGTATTACGTAATATCAGTGA

CGCGTTGAGTAATCAGAGCTTGACGCTAGTAGACGTACCAGGGGGGACGATGGTACTGCCCGGCGGAGAT

TACGCCCTGGCGGTTTATATCCTCGCCGGTATCGCAGCATTCCTCGCCGTCGTCTGCCTCGCTCTCGTCA

TCGCTTTCTTCATCAGGAACCGAACGTTGAATCGCCGCATCGAAGCTCTCTCAACCAAGTATGATACTAT

GGACACTGAGCCGACCCACACCACAGTTGAAGGGCTAGGCCTTAACAAATACGCTACCGCTCCCAACCCC

TTCAGCATCGAACCGAATATCAAAGCACCTAACTTCGACACTATTAGTGAAGCATCCGATGACCTGATCG

GCATCGAAGACATGGAACAGTTCGGAGACGACTACTTCCCGCCCGAAAACGAGATTGCAAAYCCGGCTTT

TGCACGTAACCCCATAGCGACCCATGGAAACACCTTTGGCTTGAACTCAACCCCCTTCAACCCCGAGGTC

GTGAACTCCCAGTTTAAAAGTTAA

AGTGTTGGACAGGGATGGAG

TGGCCTGACGGTTAGTTCAT

499 bp (2964-3462)

GCTGGCTCTGGCTAATATCG

CAAACTGTTGGACACCATCG

203 bp (3549-3751)

>XM_022967434 (ABCC2)

CCTAGTCGGATCGCAGGACTATTTAATATTTAGTTTGCCGTAACAGCTCTCATTAAACTAACGTTGTTGT

AGCAATGTTAATATAAAATTAATTAAAGCAATTGTGCAAATGTATAGTTGTAGGACAAAGTGTATGTGTA

ATATTTAATTTAAATAAACTCATAATGGACAAATCAAATAAAAATACCGTTTCGAACGGCACGGGCGTGG

GCGCACCAAAAGAGAGAGTAAGAAAAAAACCAAACATATTTTCGCGTATATTTGTTTGGTGGATATTCCC

TGTGCTCATTACTGGTAACAAGCGAGATGTTGAAGAAGACGATTTAATTGTTCCTAGTAAAAAGTTTAAT

TCTGAAAGACAAGGAGAATATTTTGAAAGATATTGGTTCGAGGAGGTATCAATTGCTGAGAGGGAGAACA

GAGATCCTTCACTATGGAAAGCTATGCGCCGCGCTTACTGGCTGCAGTACATGCCGGGAGCTATCTACGT

GCTGATCATTTCTGCATTAAGGACAGTTCAGCCATTGCTGTTCTCTGAACTCCTGTCCTACTGGTCAGTG

GACAGTGAAATGTCGCAGCTGGATGCTGGTCTCTATGCCCTAGCTATGTTGGGGATCAACTTCATCACCA

TGATGTGCACGCATCACAACAACTTGTTTGTCATGCGGTTCAGTATGAAAGTCAAAATCGCCGCCTCGTC

ACTCTTATTTAGAAAGTTGCTCCGCATGAGTCAAGTGTCAGTTGGCGATGTCGCAGGTGGAAAGCTAGTG

AACTTGCTGTCAAACGATGTCGCAAGGTTTGACTACGCATTCATGTTTCTGCACTACCTGTGGGTGGTAC

CCCTGCAAGTCGGAGTTGTCCTGTACTTTGTATACGATGCTGCTGGATGGGCGCCATATGTCGGTCTCTT

TGGAGTCATCATATTAATCATGCCACTTCAAGCTGGTCTAACAAAACTCACGGGCGTTGTGAGGCGGATG

ACAGCTAAGAGGACTGACAAAAGAATTAAGCTTATGAGTGAAATTATCAACGGTATACAGGTCATCAAAA

TGTACGCTTGGGAGAAACCCTTCCAGTTGGTGGTGAAGGCGGCGCGTGCTTATGAAATGAGTGCCCTTAG

GAAATCTATCTTCATCAGGAGCATGTTCCTTGGTTTCATGTTGTTCACAGAACGAAGTGTCATGTTTTTG

ACTGTGCTTACATTAGCTTTGACTGGAAACATGATTAGTGCCACTTTGATTTATCCCATCCAACAGTACT

TCGGTATTATTACAATGAATGTTACTCTCATCTTACCAATGGCGTTTGCAAGTTTCTCTGAGATGTTGAT

ATCCTTGGAACGTATTCAGGGATTCCTCCTTTTGGACGAGCGTTCAGACATTCAAATTACCCCTAAAGTG

AATGGTGCTGGAAGTAAATTGTTCAACAATTCCAAGAAGGAGGGAGGTCTCGAAACTGGCATTGTTCTGC

CAACAAAATACTCACCTACCGAAGCGAACCTTGCAAGACCCATGCAGGATGAGCCTAACATGGCTGACTA

CCCTGTGCAACTTAACAAAGTGAATGCGTCCTGGGCGGACCTCAACGAAAGCAAAGAAATGACACTCAAA

AATATGTCTTTACGTGTTCGCAAAAATAAATTGTGCGCTATCATTGGACCTGTGGGATCAGGAAAGACGT

CTCTTCTCCAGCTCCTTTTAAGAGAGTTGCCAGTGACTAGCGGGAATCTCAGCATATCTGGTACCGTGTC

CTATGCTAGTCAGGAACCTTGGCTGTTCCCAGCTACTGTGCGGGAGAACATTCTCTTTGGTTTGGACTAC

AATGTCGCCAAATATAAAGAGGTTTGCAAAGTCTGCTCATTACTGCCAGACTTTAAACAGTTCCCGTACG

GTGACTTGTCGCTGGTAGGAGAGCGAGGTGTATCACTGTCTGGTGGTCAGAGGGCTAGGATCAATTTGGC

CAGAGCTATCTACCGTGAGGCTGATATTTACTTGCTTGACGATCCTCTGTCGGCTGTAGACGCAAATGTC

GGTAGGCAATTATTCGACGGCTGTATCAAGGGATACCTCAGCGGCAAGACATGCATTCTGGTCACCCATC

AAATTCACTACCTTAAAGCTGCAGACTTTATTGTAGTCCTAAACGAGGGTTCCATCGAAAACATGGGCTC

GTATGATGAACTTATGCAAACTGGAACGGAATTCTCGATGCTGCTCTCTGACCAAGCCAGTGAGGGCTCT

GACACTGACAAAAAAGAACGGCCAGCAATGATGCGAGGAATATCAAAGATCTCAGTCAAGAGTGACGACA

ATGACGGCGAGGAGAAGGTCCAAGTATTAGAAGCTGAAGAGAGACAGTCGGGCAGTCTGAAGTGGGATGT

GCTTGCGAGGTACATGAAGTCGGTCAACTCCTGGTGCATGGTGTTCATGGCATTCCTCGTACTGGTGATC

ACGCAGGGTGCTGCCACCACTACTGACTACTGGCTTAGTTTCTGGACTAACCAAGTGGATGGTTACATAC

AAACTCTACCCGAAGGAGAAAGCCCAGATTCTGATTTGAACACACAAGTCGGTCTGCTAACAACCGGACA

GTACCTTATAGTGCACGGCAGTGTAGTATTAGCCATTATAATATTGACGCAAGTCAGAATACTTTCCTTC

GTAGTGATGACTATGCGAGCTTCGGAAAATCTTCATAACACCATTTACGAGAAATTGATAGTAGCTATAA

TGAGATTCTTCGATACCAATCCATCGGGTCGTGTCTTGAACAGATTCTCAAAAGATATGGGTGCTATGGA

CGAGCTGTTACCGCGAAGCATGTTGGAAACTGTTCAGATGTACCTGTCTCTTGCCAGTGTACTCGTGCTA

AACGCCATAGCGTTACCATGGACATTGATACCCACCACAGTGCTGATGTTCATATTTATATTCCTATTGA

AGTGGTACATTAACGCTGCTCAAGCTGTGAAACGATTGGAAGGAACAACCAAGAGTCCAGTGTTTGGAAT

GATCAACTCTACTATTTCCGGACTCTCCACCATTAGAAGTTCCAACTCTCAAGACCGACTTCTAAACTCA

TTTGATGATGCACAGAATCTCCATACCAGCGCTTTCTATACATTTTTGGGTGGTTCGACAGCTTTCGGTC

TGTACCTGGATACGCTATGTTTGATCTACCTCGGAATCATAATGTCCATCTTTATTCTTGGTGACTTCGG

TGAGTTGATACCGGTTGGTAGCGTGGGTCTGGCCGTCAGTCAGTCTATGGTGCTCACTATGATGTTGCAA

ATGGCCGCCAAGTTCACAGCCGACTTCCTGGGACAGATGACAGCCGTTGAGAGAGTACTGGAATACACCA

AGTTACCCACCGAGGAAAACATGGAGACTGGACCGACAACCCCACCAAAGGGATGGCCAAGTGCTGGAGA

GGTGACGTTCTCCAACGTTTACCTCAAATACTCTCCTGATGACCCGCCTGTACTGAAGGACTTGAACTTT

TCTATCAAGAGTGGATGGAAGGTCGGAGTAGTTGGTAGAACTGGTGCTGGCAAGTCTTCGTTGATATCAG

CTCTGTTCCGGCTCAGCGACATTACAGGCAGTATCAAAATTGATGGCCTGGATACGCAAGGGATTGCCAA

GAAGCTTTTGAGATCAAAAATATCAATAATTCCGCAAGAGCCAGTGTTATTCTCTGCCTCGCTGCGTTAT

AATCTGGATCCATTCGACGACTACAACGATGATGATATTTGGAGAGCATTGGAACAGGTGGAGCTAAAGG

AGAGTATACCGGCCCTAGATTACAAAGTGGCGGAAGGCGGCACCAACTTCTCGATGGGACAACGTCAGCT

GGTGTGTCTGGCGCGTGCTATACTCCGCTCAAATAAAATTCTCATCATGGACGAAGCTACCGCTAACGTC

GATCCTCAGACGGATGCTTTGATTCAGAAAACAATTCGTAAACAATTCGCAGCGTGCACCGTGCTCACGA

TCGCGCATCGACTGAATACCATTATGGACTCAGATCGAGTACTAGTCATGGACCAGGGAGTGGCCGCGGA

GTTCGACCACCCCTACATCTTGCTGTCTAACCCCAACAGCAAGTTCTCCTCAATGGTGAAAGAAACGGGC

GACAACATGTCCAAGATATTGTTCGAAGTAGCCAAAACAAAATATGAAAGTGATGCCAAAACCGCTTAGT

AACAAATAATAAATTTTTAGTTTGTAATAGATATAGGTAGTTGTAATTGTAATATAGTTAGATAAGTAAA

TTTATTTATTTTTACACAATGACAA

TGCCACTTCAAGCTGGTCTA

GAATGTCTGAACGCTCGTCC

453 bp (931-1383)

AACGTCAGCTGGTGTGTCTG

CAGCAAGATGTAGGGGTGGT

245 bp (3841-4085)

>JN687588 (ALP1)

ATGGGGTTGTTTAGCTTGTTTTCATTCATCACTGCCGCCATATTACTGGGATGTGTTCTCGGTGACCATT

ATCATCCGTCCGCGCCCGGCAGTCGCACTAGCTCAGCAAAGCGCGAAGAAATGGATCCCAAGTTTTGGAA

TGACAAGGCGCAGGCAGGCATCCAGGCTCGCCTCTCACAGCTGCAGGCTGCCAACAGGGCGCGCAACGTG

GTCATGTTCCTGGGCGACGGTATGTCTATGCCCACGATCTCTGCGGCGCGCGCACTGCTCGGCCAGCGCC

GCGGAGACACCGGCGAGGAGGCTGAGCTGACGTTCGATACTTTTCCTACTGTTGGTTTAATAAAGACGTA

CTGCGTGGACGCTCAGGTAGCTGACTCCGCCTGTAGTGCCACTGCATACTTGTGCGGAGTGAAAACGAAT

TACGGTGTCCTTGGCGTGAACGCAGCTGTTCCGCGAACTGACTGCGAGGCCTCGGTGGACAAGAGCACGC

ACTTGCAGTCTATCGTCGACTGGGCTCTGGCGGACGGACGAGACACCGGTATCGTGACAACAACGCGAAT

TACACATGCATCTCCTGCTGGCGCGTATGCTAAGACTGCTGATCGGTATTGGGAAAGCGATGCAGACGTT

AAGAAAGCCGGCTTCGACACCGACCGCTGCCCCGACATTGCTCACCAGCTCATACACAACCACCCTGGAA

ACAAACTAAAGGTTATTTTTGGTGGAGGTAGAGCGAACTTTTTGCCCAACTCTATCCGCGATGATGAACA

AACGTTTGGCAGCCGAACTGATAACCGGAATTTGATCCAAGAATGGCAGCAGGATAAAGTGGACCGTAAT

ATTAAACACGAATACATTTGGCATCGTGAGCAATTAATGCGTGCTAAAAACGATTTGCCAGAATACATGT

TGGGCTTGTTCGAGAGTGGTCATATGCAATATAATATGCTAGCTAACCAAACAACAGAGCCTACACTGGC

TGAAATGACTGAGGTGGCAATCCGATCTCTAAGTAGAAACGAGAAGGGCTTCTTCTTGTTCGTGGAAGGT

GGTCGCATCGACCACGCGCACCACGACAACTTCGTAGAGCTGGCGCTGGACGAGACGCTGGAGATGGACA

AGGCTGTGAAACGCGCCGCCGAACTGCTCTCTGAGGAAGACTCGCTTATTGTGGTTACAGCGGATCACTC

CCACGTCTTGGCTTACAATGGTTATGCACCACGTGGAAATGACATCTTGGGCACTTCAGTGTTTTATGCA

AATTACACATGGGATGGCATGCCTTACATGACACTATCATACACCAATGGACCAGGCTTTCGTCATCATG

TCAACGATGGCCGACCCAATGTGACCGATGAAGAAAATTATGGTACGGTAAACTGGCATTCACCTGTTGA

CGTACCGCTGAGTTATGAAACTCACGGTGGAGACGAAGTAGTGGTGTTTGCGCGCGGGCCGCACCACTCC

ATGTTCACGGGGCTGTACGAGCAGAGCCAGCTGCCGCACCTCATGGCGTACGCCGCCTGCATCGGCCCCG

GCAGACACGCCTGCAGCGCCGCCACGCATGCGCTGGCACAGCCAGTCCTGCTACTCACTCTCTTTATTCT

GTTAGCTTCCTTTGGAGTAAAATGA

AAGCGCGAAGAAATGGATCC

TCGCGTTGTTGTCACGATAC

450 bp (109-558)

CGAATACATTTGGCATCGTG

GGATTGCCACCTCAGTCATT

155 bp (849-1003)

>AF320764 (APN)

TCACTCATTTGGTACCGTTGGGCAACCATGGGTACCAAAATGTTGGTTCCCGCTGTGCTTTGCGTTCTTC

TGGGATTTGCTGCTGCCACTCCCTTAGAAGACTTTCGGTCAAATTTGGAGTTCCATGACTATTCGTCAAA

TGTTGCTGATCCAGCTTACCGTCTCCGTCCAAATGTGTACCCTACTGATGTCAAAGTAAATCTAGAGAAT

ATAGACCTTGAAGGAGCTCGCTTCACAGGATCAGTGGAGATGATCGTTATTGTAAGAGAAAACGATTTAG

AGCAAATCTCCATGCACCAGAACAATCTCTTTGTCACCAGAGTCAATGTTGTTAACAACACAAATGGTGA

AAATGTTCAACTGAGGTCCCCTGATCCCTTTACCTATGACAACTATTATGAACTCCTTCACCTTCATTTC

CACCTACCCATCGTTGCCGGCTCTTACACCATTACTATCGACTACAGAGGCGTCATCAACACAAACCCTG

TCGACAGAGGCTTCTACAGAGGTTACTATTACTACGAAAATACTCGCAGGTACTACGCCACCACACAGTT

CCAACCCTACCATGCCAGGAAAGCCTTCCCTTGTTTCGACGAGCCCCAGTTCAAGTCCCGTTACACAATT

TCCATCACCCGCCCCGACACTCTTGGCCCGTCTTACTCCAACATGGCCATCAGTTCTACCGAAGTTATCG

GCAATAGCGTTCGCGAAACATTCTACCCAACTCCCATCATTTCTGCCTACCTAGTGGCTTTCCATGTTAG

TGATTTCGTGCCCACTGTTTCTACATCTACCGCTCCTAGACCATTCAGTATTATCTCTCGCCGAGGAGCG

ACAGACCAACACGCATATGCTGCCGAAATCGGTGTGGAAATAACCAACCAGTTAGATGACTACCTTGGCA

TTGAGTACCATGACATGGGACAAGGGCAAATTATGAAGAACGATCATATTGCGCTGCCTGACTTCCCGTC

TGGTGCTATGGAAAACTGGGGAATGGTTAACTACAGAGAGGCTTACCTTTTATACGACCCTGCTAACACC

AACTTGGTCAACAAGATATTCATTGCAACCATCATGGCTCATGAACTGGGACACAAATGGTTCGGTAACC

TGGTCACCTGTTTCTGGTGGAGCAACCTTTGGCTAAACGAATCTTTTGCTAGCTACTTCGAATACTTTGC

TGCGCACTGGGCTGATCCAAAACTTGAATTAGCTGATCAATTCATCGTTGACTACGTGCACAGTGCCCTC

AATGCAGACGCGAGTCCCTCGGCTACTCCTATGAACTGGGAAGAGGTTGCAGACAATCCCACAATAACGC

AACACTTTAGTACTACCAGCTACGCCAAGGGAGCTTCTGTTCTTAGAATGATGGAGCATTTTGTTGGACC

AAGAACCTTCCGTAATGCGCTTAGACATTATTTGCGGGACAACGCCTACGGCATCGGTAACCCATCCCTG

ATGTATCAAGCATTTAATAAGGCCATCGCTGAAGATCATACATTTTTAAGTGATTTCCCGAATATCAACT

TTGGGAATGTATTCGACAGTTGGGTTCAAAACCGTGGTTCTCCAGTCGTGGAAGTCACTCGTAACCCAGA

AACTGGTGTTATTGTTGTTGAACAAAAACGTTACCAACTTTCTGGAGAACCTCCCACCCAAACTTGGGAG

ATCCCCCTCAGTTGGACCGAGCAGAAACATTTAGACTTCAGCTCGACTAAACCCAGGCAATTGTTAAATG

TAACGTCTACGGCTCTTCTAAGTGAGCCAGGAGACAACTTTGTCATATTTAACATTCAACAGTCTGGACT

GTACCGTGTCAAATACGATGAAAACAACTGGAGAGCACTTTCATCGTACCTGAACAGCAACAACAGGGAA

CGAATTCACAAACTGAACAGAGCTCAGATTGTCAATGATGTGCTGCACTTCATTCGTTCCGGGGATATCG

ACAGGACTATTGGCTTCGAAGTTATTGATTTCTTAAGAAGCGAGACTGACTACTACGTATGGAACGGTGC

TCTAACTCAGCTTGACTGGATCCGACGTCGGTTGGAGCACATGCCCAGAGCTCATGAAGCGTTCACTAGC

TACTTACGTGGTCTTATGAACAATGTTATCAACCACCTCGGATACAACGAAGGCCCCAACGACTCTACTT

CCACAATCCTGAACAGAATTCAGATCTTAAACTACGCCTGCAACATCGGGCACAGCGGTTGCGTTTCTGA

CAGTTTGCAGAAATGGAACGATTACAAAGAAAACAACGAACCGGTGCCTGTGAACCTTCGTCGCCATGTA

TATTGCACTGGTCTTCGTGAGGGTGATAGATCTGACTATGATTTCCTGTTCAATGCATACAATTCTTCAG

AAAATGCTGCTGATATGGTGATCATGCTTCGAGCTCTTGCTTGCACCAAAGATCTTGATGCACTTGGACA

TTACTTGCAAGAGAGCATGTACAACGATAAGATTCGTATCCACGACCGTACAAACGCCTTCAGTTTCGCT

TTGCAAGGAAACCTGGAAAATGTACAATTCGTTAGCCTCTTCCTTCAAAATAACTACGATACTATCAGAA

CTACATATGGTGGTGAAGCTCGTCTTACCCTCTGCGTCAACGCCGTCGCCGCCTTCCTGAATACGTTCCC

AGCAATAACTCAATTCCAGACGTGGGCCTATGGATCCCAAGTCGGCTTAGCCGGGTCGTTCAACGCCGCT

GTGAATGTAGTCAACAGTGCCATGGCCAATCTAGACTGGGGTAGCAATAATGCGCTTGAAGTCTTCAATT

TCGTTTCTGTTAGAAGCAGCTCCCCTACCATCTTCGCTTCATCACTCCTCATCCTCGCAGCTATGCTCAT

TCAGCTGTACCGTTAAATTATAATAAAATAATCTAACCAAATATATACTAATAAATCATCTAATAACTTT

CTTATGAGATTCCTGTGGAGTTATTAAATGCTGTCTAAATAATAAATGAAAAATTAAACTTTAGAAATAG

TTTTCCACATTTACATGTTGTTTTCGTCTTGTACGTATTTAATTATAATAAAATATTTTTTGTACATAAA

TTTCTTAAAAAAAAAAAAAAAAAAA

TACTACGCCACCACACAGTT

AAGTCAGGCAGCGCAATATG

434 bp (541-974)

CATCAACACAAACCCTGTCG

TGATGGCCATGTTGGAGTAA

209 bp (474-682)
